# Propofol mitigates brain injury and oxidative stress, and enhances GABAA receptor α1 subunit expression in a rat model of lithium chloride-pilocarpine induced status epilepticus

**DOI:** 10.55730/1300-0144.5670

**Published:** 2023-05-25

**Authors:** Lei LI, Xiu LIU, Juan DU, Wangyan YANG, Runqiao FU, Yunfeng LI, Wei ZHAO, Henglin WANG

**Affiliations:** 1 Department of Anesthesiology, Beijing Chuiyangliu Hospital Affiliated to Tsinghua University, Beijing, China; 2 Department of General Surgery, Peking Puren Hospital, Beijing, China; 3 State Key Laboratory of Toxicology and Medical Countermeasures, Beijing Institute of Pharmacology and Toxicology, Beijing, China; 4 Department of Anesthesiology, China-Japan Friendship Hospital, Beijing, China; 5 Department of Anesthesiology, The Sixth Medical Center of Chinese People’s Liberation Army General Hospital, Beijing, China

**Keywords:** Propofol, status epilepticus, antioxidant activity, GABAA receptor a1 subunit

## Abstract

**Background/aim:**

Propofol is a positive allosteric modulator of GABAA receptor (GABAAR) and has potent antioxidant activity. The aim of this study was to investigate the effect of propofol on damage to the cerebral cortex and hippocampus in a lithium chloride (LiCl)-pilocarpine animal model of status epilepticus (SE).

**Materials and methods:**

Adult male Sprague Dawley rats were injected with LiCl-pilocarpine to induce SE. They were then randomized and injected 30 min later with vehicle saline (SE+saline), propofol (SE+PPF, 50 mg/kg), Diazepam (SE+DZP, 10 mg/kg), Scopolamine (SE+SCOP, 10 mg/kg), or MK-801 (SE+MK-801, 2 mg/kg). Another group of rats received saline only and served as the naïve control (BLK). The levels of superoxide dismutase (SOD), glutathione (GSH) and malondialdehyde (MDA) in the serum, cortex and hippocampus were analyzed 2 and 24 h posttreatment. The degree of tissue damage in the cortex and hippocampus of individual rats was assessed 24 h posttreatment, together with expression of the GABAAR α1 subunit.

**Results:**

The propofol group showed reduced levels of tissue damage in the cerebral cortex and hippocampus, decreased levels of MDA, and increased levels of GSH compared to the SE+saline group. No changes in SOD level were observed in serum and tissue samples from the cortex and hippocampus of SE+saline rats. Immunohistochemistry and Western blot assays showed that propofol treatment significantly increased the expression of GABA_A_R α1 subunit in the cortical and hippocampal tissues of SE rats.

**Conclusion:**

Propofol treatment protected against SE-induced tissue injury in the cortex and hippocampus of rats. This was due at least in part to its antioxidant activity and to its induction of GABAAR α1 subunit expression in the brain.

## 1. Introduction

Status epilepticus (SE) is one of the most commoncomplications of chronic disease in the central nervoussystem (CNS). It can result from brain injury caused by avariety of etiological factors, and can be life-threatening if the patient is not treated. Approximately 25% of epilepticpatients do not respond to first- and second-line drugtherapies and may develop refractory epilepsy, which isa serious social and public health issue. Therefore, theearly detection and drug treatment of patients with SEis critical [1].

Currently, the pathogenesis of epilepsy remains unclear. A previous study suggested that oxidative stress is crucial for the development and progression of epilepsy [2]. Oxidative stress can promote the production of reactive oxygen species (ROS) in the brain, which in turn causes dysfunction of the mitochondrial respiratory chain, exhaustion of endogenous antioxidants, lipid peroxidation, tissue damage and epilepsy [3,4]. Superoxide dismutase (SOD), and glutathione (GSH) are the major indirect free radical scavengers, while malondialdehyde (MDA) is an indicator of lipid peroxidation and oxidative stress [5]. Pathogenic factors may therefore alter GABA release, GABA_A_R internalization and signaling during the development of epilepsy [6,7]. Hence, scavenging of ROS may attenuate ROS-mediated brain tissue damage and GABA_A_R dysfunction, thereby providing possible therapy for SE.

Propofol is a positive allosteric modulator of GABA_A_R and has been widely used in the clinic as a short-action anesthetic drug [8, 9]. Furthermore, propofol has neuroprotective and antioxidant activity in animal models of ischemia-reperfusion brain injury via the reduction of brain oxygen consumption and lipid peroxidation [10,11]. Propofol can also inhibit cortical activity by modulating opening of the GABA_A_R-gated chloride channel [12,13]. A previous study showed that GABA_A_R a1 subunit expression in the cortex and hippocampus is downregulated during the process of SE [14]. However, there is no information on whether treatment with propofol can modulate oxidative stress and expression of the GABA_A_R a1 subunit in the cortex and hippocampus during SE.

In the current study, we used a rat model of LiCl-pilocarpine-induced SE [15] to investigate the effect of propofol on oxidative stress, tissue damage in the cortex and hippocampus, and GABA_A_R a1 subunit expression. Diazepam, MK-801 (an NMDA receptor antagonist) and scopolamine (an anticholinergic drug) were used as comparison interventions.

## 2. Materials and methods

### 2.1. Animals

Adult male Sprague-Dawley (SD) rats aged 6 to 8 weeks and weighing 200–220 g were obtained from the Animal Experiment Center of the Academy of Military Medical Sciences. They were housed in a specific pathogen-free facility at a constant temperature of 23 ± 1 °C and a 12 h day/night cycle, with unlimited access to water and food. All procedures and tests were conducted between 8:00 am and 12:00 am. The experimental protocols were established according to NIH publication No. 86-23 (1996 revision) and approved by the Institutional Committee on Animal Care and Use of Academy of Military Medical Sciences (no. IACUC.20094).

### 2.2. Reagents and drugs

Lithium chloride, pilocarpine, diazepam, scopolamine, and MK-801 were purchased from Sigma. Propofol was purchased from AstraZeneca. Rabbit polyclonal antibodies against mouse and human GABA_A_ receptor a1 subunits were purchased from Alomone. Horseradish peroxidase (HRP)-conjugated goat anti-rabbit IgG and 3,3-Diaminobenzidine (DAB) were purchased from ZSBIO. Protein BCA assay kit, Western Blot Stripping Buffer, skimmed milk powder, nitrocellulose membrane, and Super ECL Plus were purchased from Beijing Applygen Technologies. Wide-range prestained protein marker (molecular weight standard: 6–200 kd) was purchased from New England Biolabs. Other assay kits were purchased from the Nanjing Jiancheng Bioengineering Institute.

### 2.3. Induction of SE by pilocarpine and preparation of tissue sample

**Figure 1:**
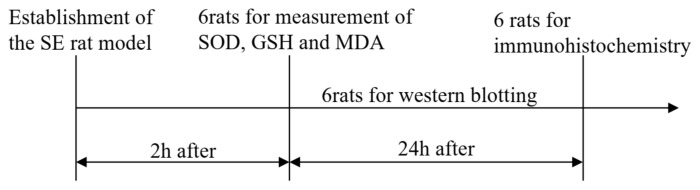
Schedule and test order for rats.

A rat model of SE was induced by injecting LiCl-pilocarpine and was evaluated as described previously [15,16,25]. Briefly, rats were randomized into six groups (18 per group) as follows: naïve control (BLK), SE seizure (SE+saline), 50 mg/kg propofol (SE+PPF), 10 mg/kg diazepam (SE+DZP), 10 mg/kg scopolamine (SE+SCOP), and 2 mg/kg of the nonspecific NMDA receptor antagonist dizocilpine (SE+MK801). At 20–24 h before the experiment, all rats except those in the BLK group were injected intraperitoneally (i.p) with LiCl at a dose of 3 mEq/kg (127 mg/kg). On the day of the experiment, rats were injected i.p with pilocarpine at a dose of 30 mg/kg (maximum dose, 60 mg/kg) until SE was successfully induced. The SE seizure was quantified according to the rating scale adapted from Racine. In the present study, recurrent seizures greater than or equal to Racine stage 4 and 5 and that lasted for 30 min were used for further analysis. Diazepam (10 mg/kg) was administered intraperitoneally 1 h after the onset of SE to terminate behavioral seizures, were excluded from our subsequent experiments. At 30 min post SE, the different groups of rats were injected i.p with propofol, SCOP or MK801 with the doses described above. The BLK and SE groups were injected with vehicle saline. Two h after treatment, six rats from each group were randomly selected and peripheral blood samples obtained by orbital bleeding and serum samples prepared by centrifugation. Rats were sacrificed following anesthetization with S-ketamine (100 mg/kg i.p.) and xylazine (15 mg/kg i.p.). The cerebral cortex and hippocampus tissues were removed, immediately frozen in liquid nitrogen, and stored at −80 °C for later measurements. At 24 h post treatment, another six rats from each group were subjected to the same procedure described above. The remaining six rats from each group were anesthetized at 24 h posttreatment and perfused transcardially with 4% paraformaldehyde (PFA, 50 mL per rat). The cerebral cortex and hippocampus tissues of individual rats were excised and paraffin-embedded (Figure 1).

### 2.4. Hematoxylin-Eosin staining

The morphology of the cerebral cortex and hippocampus tissues was evaluated by hematoxylin-eosin (H&E) staining. Briefly, tissue sections (5 μm) were stained with H&E and scanned using a computer-assisted, light BX-50 General Biological Microscope (Olympus, Japan). After the regions (CA1-3 pyramidal cell layers) were outlined, 10 areas from the hippocampus of each rat were selected and the gray values measured. Intensity measurements were represented as the mean number from a 256-gray scale using NIH Image 1.59 software.

### 2.5.Measurement of SOD, GSH, and MDA by ELISA

One day after treatment, the remaining six rats from each group were sacrificed and their cerebral cortex and hippocampus tissues were removed. Individual tissues were weighed and homogenized with lysis solution, followed by centrifugation at 12,000 g for 30 min. The tissue lysates were collected and their protein concentration determined using a BCA protein assay kit (Pierce, USA). Serum samples were also prepared and the SOD, GSH and MDA levels in individual serum and brain tissue samples were determined by ELISA using standard assay kits according the manufacturer’s instructions (Nanjing Jiancheng Bioengineering Institute). The results were expressed as micromoles per milliliter of serum (μmol/mL), or as micromoles per milligram of wet tissue protein (μmol/mg prot) [17,18].

### 2.6. Immunohistochemistry analyses

One day after treatment, 6 rats from each group were perfused transcardially with 4% paraformaldehyde. The brain tissues were removed, postfixed overnight with cold paraformaldehyde, and then transferred onto 30% sucrose in phosphate-buffered saline (PBS) until they sank to the bottom. They were then embedded in OCT using dry ice and 2-methylbutane. Coronal cryostat tissue sections (30 μm) were prepared using a freezing microtome and stored at −20 °C. The sections were washed with 0.01 M PBS and treated with 3% H_2_O_2_/methanol for 10 min. After washing, the sections were blocked with 10% goat serum in TBST at 37 °C for 1 h and incubated with rabbit anti-Iba1 (1: 1000) or anti-GABAAR a1 (1: 200, Alomone, Israel) at 4 °C overnight. After washing, the sections were incubated with biotinylated goat anti-mouse/rabbit IgG at 37 °for 10 min and then incubated with HRP-conjugated streptavidin at 37 °C for 15 min, followed by DAB staining and counterstaining with haematoxylin. Three regions each from the cortex and hippocampus sections of each rat were selected for microscopic imaging. Histological changes were analyzed using Image-Pro plus 5 imaging software, with positively stained cells counted using a microscope connected via CCD camera to a PC monitor. The regions were outlined and the surface areas measured at a magnification of 200 ×. An estimate of the cell number was then made using the Abercrombie correction method [19].

### 2.7. Western blotting

The relative levels of GABA_A_R a1 expression in individual frontal cortex and hippocampus tissue samples (n = 6 per group) were determined by Western blot analysis. Individual samples (50 μg per lane) were separated by sodium dodecyl sulfate polyacrylamide gel electrophoresis (SDS-PAGE) on 8% gels and transferred onto nitrocellulose membrane. The membranes were blocked for 2 h with 5% dry skim milk in TBST and then incubated overnight at 4 ⊠ with anti-GABA_A_R a1 (1:400) and anti-β-actin (1:400) antibodies. After washing, the bound antibodies were detected with HRP-conjugated goat anti-rabbit or rabbit anti-goat secondary antibodies (1: 2000, Pierce, Rockford) for 1 h and visualized using an enhanced chemiluminescence (ECL) detection system. The levels of target protein relative to β-actin were analyzed using the FluorChem™FC2 gel imaging system and ImageJ software [20].

### 2.8. Statistical analysis

Data are expressed as mean ± standard error of the mean (SEM). Differences between groups were analyzed by one-way ANOVA and post hoc Dunnett’s t-test, and by Student’s t-test using GraphPad Prism, Version 5.0 (GraphPad, San Diego, CA, USA). p-value of <0.05 was considered statistically significant.

## 3. Results

### 3.1. Cortex and hippocampus morphology after treatment with drugss

**Figure 2:**
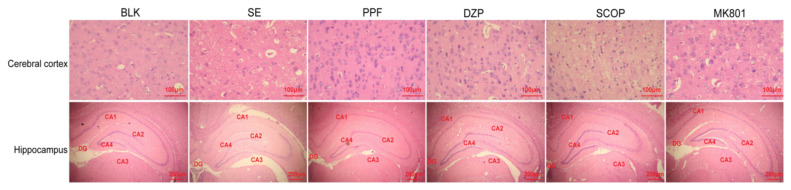
The cerebral cortex and hippocampus tissue damages in rats. Adult SD rats were injected with LiCl-Pilocarpine to induce SE. The rats were randomized and treated with vehicle (SE+saline group), propofol (SE+PPF group), diazepam (SE+DZP group), scopolamine (SE+SCOP group), or dizocilpine (SE+MK801 group). A control group of rats received saline (BLK group). At 24 h post treatment, their cerebral cortex (prefrontal and parietal cortex, showing labeling in layers II/III and V) and hippocampus tissue (CA1) damages were examined by histology after H&E staining. Data were representative images of the cortex (magnification × 200, the top panel) and the hippocampus (magnification × 40) of each group (n = 6 per group). ^#^p < 0.05 vs. the BLK group; ^*^p < 0.05 vs. the SE+saline group.

Tissue sections from the cortex and hippocampus at 24 h posttreatment were examined by histology. Normal cortex and hippocampus morphology with uniform cell arrangement was observed in the BLK naïve group of rats (Figure 2). In contrast, damage to the cerebral cortex (prefrontal and parietal) and hippocampus (CA1) neurons with aberrant orientation, fragmentation, nuclear pyknosis and condensed cytoplasm was detected in the SE+saline group (F = 43.6, SE+saline, Figure 2). However, few changes were observed in the cortex and hippocampus of the SE+PPF, SE+DZP, and SE+MK-801 groups, with some scattered neurons exhibiting slight swelling and rupture changes (Figure 2). Damage to the cortex and hippocampus tissues in the SE+SCOP group was similar to that seen in the SE+saline group (F = 43.6, Table).

### 3.2. SOD, GSH, and MDA levels in the serum and brain of individual rats at 2 h and 24 h posttreatment

**Figure 3:**
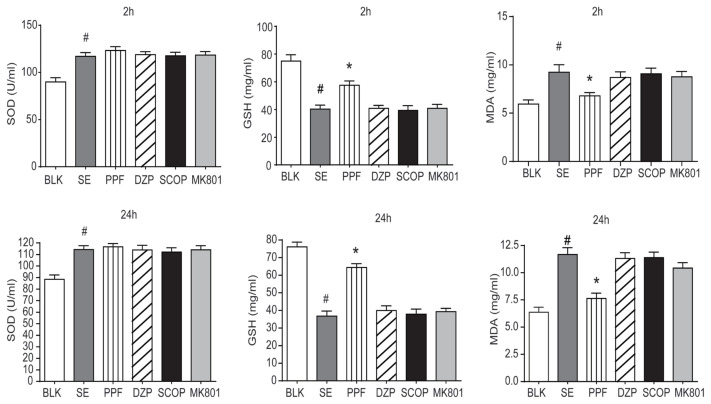
SOD, GSH, and MDA levels in the serum of individual rats at 2 h and 24 h post treatment. The levels of SOD, GSH, and MDA in serum of individual rats at 2 h and 24h post treatment were determined by ELISA. Data were expressed as the means ± SEM of each group (n = 6). ^#^p < 0.05 vs. the BLK group; ^*^p < 0.05 vs. the SE+saline group.

**Figure 4:**
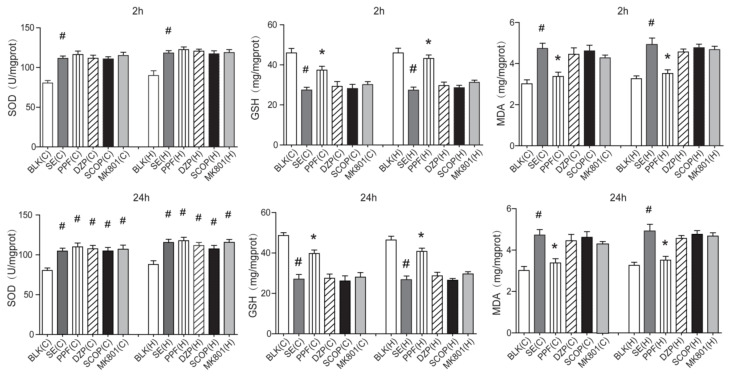
SOD, GSH and MDA levels in the brain of individual rats at 2 h and 24 h post treatment. The levels of SOD, GSH, and MDA in the cerebral cortex and hippocampus tissue sample of individual rats at 2h and 24 h post treatment were determined by ELISA. Data were expressed as the means ± SEM of each group (n = 6). ^#^p < 0.05 vs. the BLK group; ^*^p < 0.05 vs. the SE+saline group.

LiCl-Pilocarpine can induce oxidative stress and MDA production, thereby exhausting antioxidant GSH and SOD. To determine the potential mechanisms underlying the protective effect of propofol, the levels of SOD, GSH, and MDA in the serum and brain tissue of SE rats were examined at 2 h and 24 h posttreatment. The levels of serum SOD (2 h, F = 9.8; 24 h, F = 8.9) and MDA (2 h, F = 19.9; 24 h, F = 18.1) were significantly higher in the SE+saline group compared to the BLK group, whereas the level of GSH (2 h, F = 6.1; 24 h, F = 43.75) was lower in the SE+saline group (p < 0.05 for all; Figure 3). The level of serum MDA in the SE+PPF group was significantly lower compared to the SE+saline group, whereas serum GSH was higher and serum SOD was the same. The levels of serum SOD, GSH and MDA did not change significantly in the groups treated with diazepam, scopolamine or dizocilpine compared to SE+saline. Furthermore, similar levels of SOD (C: F = 13.9; H: F = 9.6), GSH (C: F = 13.8; H: F = 22.9) and MDA (C: F = 9.1 H: F = 12.5) in the cortex and hippocampus tissues were observed in the different groups (Figure 4).

### 3.3. GABA_A_R a1 subunit expression in the cortex and hippocampus tissues following treatment

**Figure 5:**
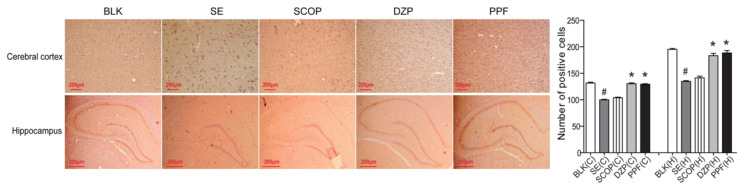
Immunohistochemistry of GABA_A_R α1 subunit expression in the cerebral cortex and hippocampus tissues. The levels of GABA_A_R α1 subunit expression in the cerebral cortex and hippocampus tissues of individual rats were characterized by immunohistochemistry and the numbers of GABA_A_R α1+ cells in the hippocampus were analyzed. Data are representative images (magnification × 200) or expressed as the means ± SD of each group (n = 6). ^#^p < 0.05 vs. the BLK group; ^*^p < 0.05 vs. the SE+saline group.

**Figure 6:**
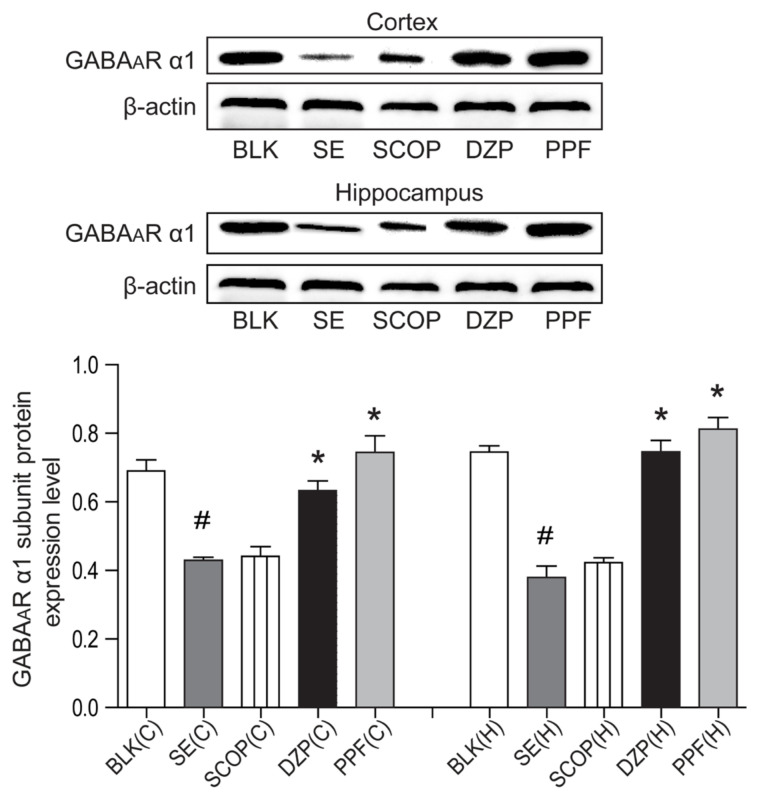
Western blot of GABA_A_ receptor a1 subunit expression in the cerebral cortex and hippocampus. The relative levels of GABA_A_R α1 subunit to the control β-actin expression in the cerebral cortex and hippocampus tissues of individual rats were characterized by Western blot. Data are representative images or expressed as the means ± SEM of each group (n = 6). ^#^p < 0.05 vs. the BLK group; ^*^p < 0.05 vs. the SE+saline group.

GABA_A_R a1 expression in the cortex and hippocampus tissues of the SE and SCOP groups was reduced compared to the BLK group (Figure 5). This reduction was attenuated by treatment with propofol or diazepam. Quantitative analysis revealed that the number of GABA_A_R a_1_ positive cells in the cortex and hippocampus of the SE+saline and SE+SCOP groups was significantly lower than in the BLK, SE+DZP, and SE+PPF groups (F = 15.3, Figure 5). Western blot analysis revealed similar patterns for the relative levels of GABA_A_R a1 expression in the cortex and hippocampus of the different groups of rats (F = 8.4, Figure 6).

## 4. Discussion

This study investigated the potential protective effects of propofol against brain injury induced by LiCl-pilocarpine in a rat model of SE. Similar to diazepam, treatment with propofol significantly mitigated damage to the cortex and hippocampus in SE rats. These results support the notion that activation of GABA_A_R protects against oxidative stress-induced neuronal injury [21,22]. Our novel data suggest that propofol may therefore be valuable for treatment against SE and other oxidative stress-related neuronal degeneration diseases.

MK-801 is an NMDA receptor antagonist that induces hyperlocomotion partly by activating dopamine neurons in the prefrontal cortex and hippocampus. Scopolamine, a cholinergic receptor blocker, acts by blocking muscarinic acetylcholine receptors on cholinergic neurons, thereby disinhibiting these neurons and allowing them to activate dopamine neurons. These common pharmacological tools are used to induce learning and memory deficits in many animal models. However, they had no effect on these changes, which could imply that the mechanism by which SE was induced in the secondary phase was not associated with cholinergic neurons. Further studies are required to elucidate the mechanism by which MK-801 impairs cognitive functions such as learning and memory. Moreover, allosteric modulators of GABA_A_ receptor such as benzodiazepines could progressively lose their potency as synaptic GABA_A_ receptors internalize. Such therapeutic limitations highlight the need for improved antiseizure medications to treat pharmaco-resistant seizure [23–25].

Indeed, in the present study we detected higher levels of SOD and MDA in the brain and peripheral sera of SE rats, but lower levels of GSH. Moreover, treatment with propofol was found to significantly increase the level of GSH in the brain and peripheral sera, but to reduce the level of MDA. Similar levels of SOD, GSH, and MDA were observed in the serum and cortex hippocampus tissues in the other groups, suggesting the combination of diazepam and MK801 had a stronger anticonvulsant effect than single application. Many studies have shown that pilocarpine-induced SOD activity in the hippocampus of rats did not change significantly in the cortex and hippocampus of rats. However, in the present we found significantly increased SOD activity in the cortex and hippocampus of SE rats. This may be due to a compensatory increase in SOD activity caused by intense oxidative stress following epileptic seizure, which could be a manifestation of the self-protection mechanism of oxidative injury in the brain. Therefore, it appears likely that excessive oxidative stress is both a cause and consequence of convulsive SE, with a predominance of one or the other in different clinical scenarios. Furthermore, oxidative stress is a major factor in neuronal injury and damage during the process of SE. Excessive oxidative stress can also change the levels of the GABA neurotransmitter, the GABA_A_ receptor subunit, and GAD expression in the hippocampus of epileptic rats [26,27]. Activation of the GABA_A_ receptor inhibits epileptic seizure, while temporary GABA_A_ receptor-mediated synaptic potentials enhance pyramidal cell hyperexcitability in the CA1 and CA3a hippocampal areas (but not in the CA3b and hilus), thereby contributing to the development of temporal lobe epilepsy in rats [28–29].

SE in the rat brain is consistently associated with neuronal injury in several distinct areas outside of the hippocampus, suggesting a possible role in epileptogenesis. An earlier study reported that excessive oxidative stress alters the levels of the GABA neurotransmitter and GABAA receptor expression in the hippocampus of epileptic rats. Propofol could cause the triggering of GABA_A_ receptors and subsequent time-dependent neuroprotection of primary cortical neurons, thus causing anticonvulsion effects. Drugs that inhibit GABA_A_ receptor, such as propofol, midazolam and pentobarbital, are recommended for the management of refractory status epilepticus (RSE). RSE is defined as SE that fails to adequately respond to first- and second-line antiepileptic drugs. Evidence obtained from animal models indicates that propofol reduces oxidative stress, markers of inflammation, and lipid peroxidation. In the present study, significantly reduced expression levels of GABA_A_ receptor a1 were observed in the cortex and hippocampus of SE rats. Oxidative stress could cause neuronal injury and death leading to reduced expression of GABA_A_ receptor a1 in the brain, consistent with previous observations [30–31]. These findings also indicate that reduced levels of GABA_A_ receptor a1 expression in the brain contribute to the development and progression of SE. Importantly, we found that propofol treatment attenuated the reduction in GABA_A_ receptor a1 expression in the brain of SE rats. This is an interesting finding considering the small number of studies on excessive oxidative stress, in particular on SOD action. The effect of SOD is likely to be caused by an entirely different mechanism and not because of an effect on the GABA_A_ receptor. Therefore, the therapeutic effect of propofol on SE may be due to an overall effect that is the result of several different mechanisms. Some studies have shown that propofol has greater potency at decreasing inflammation and oxidative stress compared with midazolam. Further studies are required to identify the mechanism that underlies the therapeutic effect of propofol on SE, and whether rat models of SE can be treated with other drugs such as midazolam or ketamine.

## 5. Conclusion

The present findings indicate that high levels of oxidative stress can down-regulate GABA_A_R a1 expression in the brain, thus contributing to the development of SE. Propofol treatment can mitigate this oxidative stress, thereby protecting against down-regulation of GABA_A_R a1 expression in the brain. This may be one of the factors that contributes to the positive effects of propofol in SE, although the therapeutic effect is likely to be the overall result of several different mechanisms. However, the precise mechanisms that underlie the therapeutic effect of propofol and its clinical efficacy require further study. Future studies should also establish, in a prospective manner, the level of GABA_A_R antibodies in the serum of patients with seizures or SE.

## Figures and Tables

**Table:** Measured gray values of the cerebral cortex and hippocampus tissue damages in rats.

group	The cortex	Hippocampus
BLK group	36 ± 3.17	23 ± 4.08
SE group	38 ± 5.12#	51 ± 5.11#
PPF group	17 ± 3.19	21 ± 4.93
DZP group	29 ± 4.34	97 ± 4.12
SCO group	20 ± 5.02	32 ± 5.22
MK801 group	71 ± 3.09*	28 ± 6.31*

The representative images of the cortex and the hippocampus regions (CA1–3 pyramidal cell layers) of each group were outlined, 10 areas of each rat were selected and the gray values measured. Intensity measurements were represented as the mean number from a 256-gray scale. Data were expressed as the means ± SEM of each group (n = 6).

# p < 0.05 vs. the BLK group;

* p < 0.05 vs. the SE+saline group.

## Abbreviations

SE: status epilepticus
RSE: refractory status epilepticus
GABA_A_R: gamma-aminobutyric acid subscript A receptor
MK801: dizocilpine maleate
ROS: reactive oxygen species
SOD: superoxide dismutase
GSH: glutathione
MDA: malondialdehyde
CNS: central nervous system
DAB: diaminobenzidine
PBS: phosphate buffered saline
NMDAR: N-methyl-D-aspartate receptor
